# Construction of microbial consortia for microbial degradation of complex compounds

**DOI:** 10.3389/fbioe.2022.1051233

**Published:** 2022-12-06

**Authors:** Zhibei Cao, Wenlong Yan, Mingzhu Ding, Yingjin Yuan

**Affiliations:** ^1^ Frontier Science Center for Synthetic Biology and Key Laboratory of Systems Bioengineering (Ministry of Education), School of Chemical Engineering and Technology, Tianjin University, Tianjin, China; ^2^ Collaborative Innovation Center of Chemical Science and Engineering (Tianjin), Tianjin University, Tianjin, China

**Keywords:** microbial consortia, degradation, complex compounds, plastic biodegradation, petroleum biodegradation

## Abstract

Increasingly complex synthetic environmental pollutants are prompting further research into bioremediation, which is one of the most economical and safest means of environmental restoration. From the current research, using microbial consortia to degrade complex compounds is more advantageous compared to using isolated bacteria, as the former is more adaptable and stable within the growth environment and can provide a suitable catalytic environment for each enzyme required by the biodegradation pathway. With the development of synthetic biology and gene-editing tools, artificial microbial consortia systems can be designed to be more efficient, stable, and robust, and they can be used to produce high-value-added products with their strong degradation ability. Furthermore, microbial consortia systems are shown to be promising in the degradation of complex compounds. In this review, the strategies for constructing stable and robust microbial consortia are discussed. The current advances in the degradation of complex compounds by microbial consortia are also classified and detailed, including plastics, petroleum, antibiotics, azo dyes, and some pollutants present in sewage. Thus, this paper aims to support some helps to those who focus on the degradation of complex compounds by microbial consortia.

## Introduction

**FIGURE 1:**
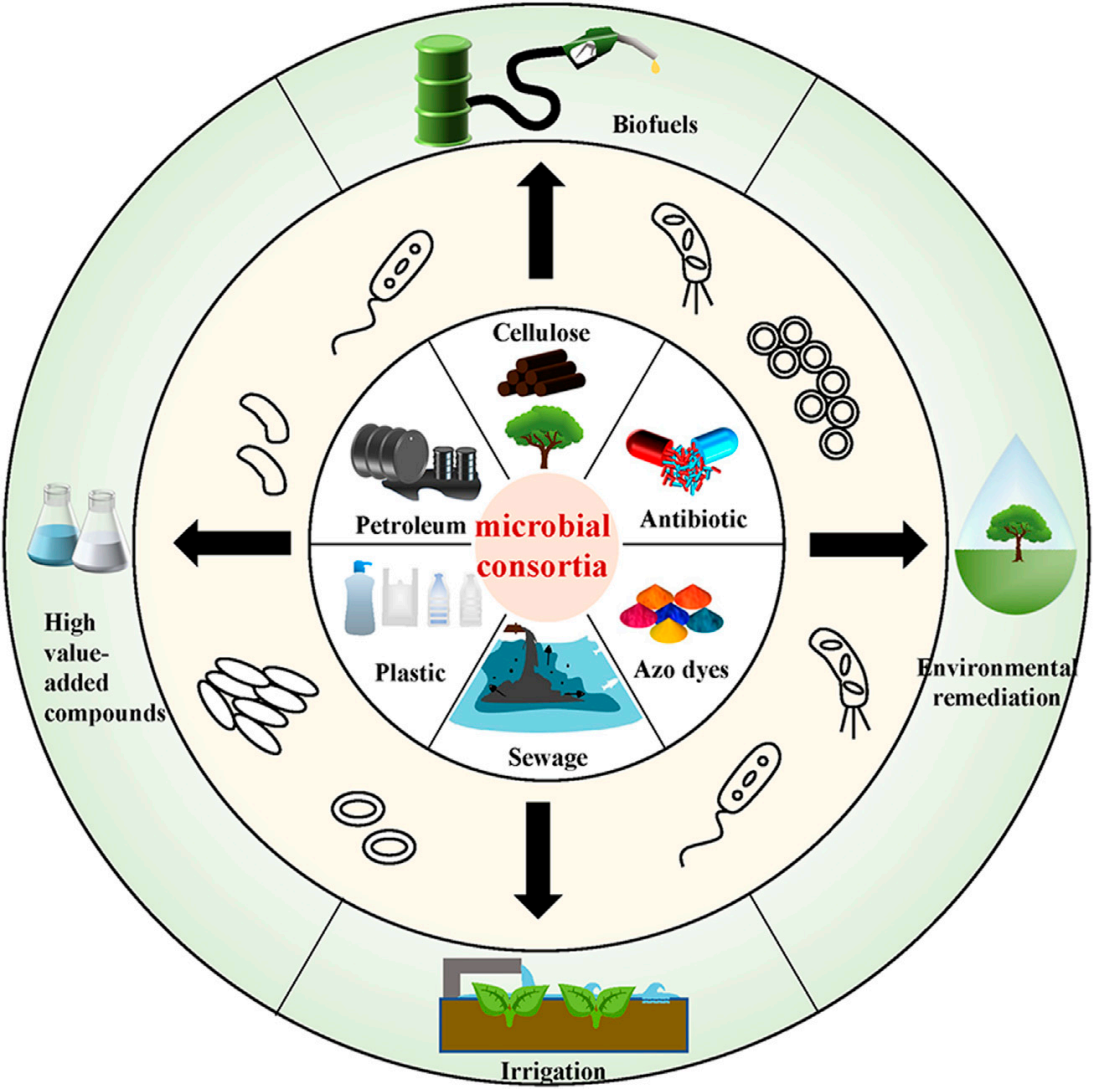
Microbial consortia bioremediation and reuse of complex compounds.

In recent years, increasingly serious pollution has been a major threat to public health, and more and more people are putting forward higher requirements for environmental restoration. Microbial environmental remediation is cleaner and more economical than the traditional burning landfill, which causes subsequent environmental pollution. The use of microbial consortia to degrade various pollutants into non-toxic or less-toxic compounds is a better option (Azubuike et al., 2016). At present, research on bioremediation by microbial consortia has practical significance, and it has already been applied in some cases. As shown in Figure 1, microbial consortia can degrade complex compounds, including plastics, petroleum, antibiotics, azo dyes, and some pollutants present in sewage. Furthermore, it also can be used in consolidated bioprocessing (CBP), which is a great solution to energy shortages. Petroleum hydrocarbons and plastics can also be used as raw materials for the production of high-value-added products. Because of the excellent degradation ability of microbial consortia for complex compounds, they are more commonly used than the single stain in environmental remediation. For example, they can well degrade the complex compounds in soil and sewage. Furthermore, shrubs and trees can be planted on the treated soil, and the treated sewage can be used as irrigation water for non-edible commercial crops (Biswas et al., 2021).

Creating an artificial microbial consortia system distributes the desired multiple catalytic enzyme expression pathways to different strains, and then co-culturing all strains to complete the task (Zhang and Stephanopoulos, 2016; Li Z. et al., 2019). The microbial consortia can degrade complex compounds that cannot be decomposed by a single bacterial system, such as starch and cellulose. Some complex compounds are difficult to be degraded due to their complex structures. However, some strains can break down these complex substrates into small-molecule sugars that can be used as carbon sources for other strains in the system (Wang S. et al., 2019; Tondro et al., 2020). For strains in the consortia, the rational division of metabolic pathways can reduce cross-reactions and thus the metabolic burden of each cell (Said and Or, 2017; Shen et al., 2020). Compared with a natural microbial consortia system, the composition of an artificial microbial consortia system is simpler, the division of labor is clearer, and it can be further modified for different target products (Qian et al., 2020; Zhang and Hong, 2020). Thus, a microbial consortium can be constructed to degrade a wide range of complex compounds precisely, which can enable the modular assembly and optimization of metabolic pathways by modulating the microbial consortia structure (Jones et al., 2017; Jones and Wang, 2018; Roell et al., 2019). Cross-feeding between bacteria can also be used to eliminate feedback inhibition and remove products or by-products, which is important for improving the degradation efficiency of complex compounds (Zhou et al., 2015). Microbial consortia also have strong adaptability to, and stability within, complex environments (Kaeberlein et al., 2002). After a variety of cells with different functions are fused, the dynamic balance is maintained through complex interactions between cells, making the entire system more adaptable and stable when facing environmental fluctuations (McCarty and Ledesma-Amaro, 2019). The synergistic development of systems and synthetic biology will provide both a thorough understanding and a rational engineering of these complicated consortia for novel applications (Song et al., 2014).

The advantages described above are inspiring more and more researchers to explore the ability of microbial consortia to degrade complex compounds. In this paper, to provide a reference for the construction of microbial consortia, those that are currently used to degrade complex compounds are summarized, and future research directions for their construction are discussed.

## Construction strategy of microbial consortia for degradation

Artificial microbial consortia systems have been developed and studied based on natural microbial consortia systems. There are usually two principles for the design of artificial microbial consortia systems: the top-down approach and the bottom-up approach. The top-down approach uses carefully selected environmental variables that force an existing microbiome (naturally occurring or inoculated) through ecological selection to perform the desired biological processes. This requires us to conceptualize the microbial consortia as a system model and determine the inputs and outputs of the system, including physical and chemical conditions, known abiotic and biological processes, environmental variables, and how operations on the microbial consortia promote or inhibit the biological processes being optimized (Lawson et al., 2019). The most commonly used method is to artificially enrich and screen functional microbial consortia. Although the conventional top-down approach offers a framework and has been widely successful for wastewater treatment and bioremediation, it often ignores processes that depend on intricate interactions between consortia members. Recent advances in synthetic biology have enabled researchers to develop bottom-up approaches and focus on engineering the microbiome’s metabolic network and microbial interactions. The general design process is to obtain the genomes of individual members of the microbiome and then reconstruct the metabolic networks. The individual populations’ reactions and metabolites can be compartmentalized and metabolic fluxes within and between populations can be simulated using optimality principles (Orth et al., 2010). These models can also simulate steady-state flux distributions over time and space. Such bottom-up tools provide a platform for rationally designing microbiomes with specific properties such as distributed pathways, modular species interactions, community resistance and resilience, and spatiotemporal organization that optimize ecosystem function and stability. Therefore, extending these designs to systems with non-model organisms of tens to hundreds of different species will require deeper insights into their metabolism and the principles governing their interactions and higher-order behavior (Lawson et al., 2019). Most microbial consortia that degrade complex compounds are constructed with the top-down approach.

When constructing microbial consortia to degrade complex compounds, one of the important issues is to select suitable chassis strains with suitable catalytic performance; whether they can coexist with other strains also needs to be considered (Jawed et al., 2019). Therefore, in the selection of chassis strains, strains with low mutation rates, non-toxic by-products, and high tolerance are generally selected. The next issue that needs to be considered is the division of degradation pathways. Long degradation pathways can be rationally divided into several strains, and different degradation pathways can be responsible for different strains (Lu et al., 2019). Although an artificial microbial consortia system can reduce the metabolic burden of cells, excessive segmentation of metabolic pathways will also lead to confusion and reduce the efficiency of mass transfer (Goers et al., 2014). In recent years, it has been discovered that the ordered spatiotemporal distribution of strains can improve the efficiency of microbial consortia to degrade complex compounds. In this way, each strain in the microbial consortia is provided with a suitable environment for degradation and a spatial position corresponding to the time sequence in the degradation pathway. Strain immobilization is a commonly used spatio-temporal distribution application, and plays an important role in promoting the biodegradation of complex compounds. It can be implemented through an ambient medium. Some researchers developed a special hydrogel as a new carrier to be used in the immobilization of artificial microbial consortia systems. This kind of hydrogel not only does not affect the material exchange of bacteria but also has a preservation effect on bacteria, which is conducive to the stability of their function. Strains with different environmental requirements in the microbial consortium can be preserved in different hydrogels, and the mixing of hydrogels does not change their individual properties (Johnston et al., 2020). The design of the culture device is also helpful to the spatio-temporal distribution and control of strains in an artificial microbial consortia system. Microfluidic technology achieves the fine regulation of different strains and improves the control of the microbial consortia system (Wang C. et al., 2019). Some researchers designed a ventilated biofilm reactor based on the gradient distribution of oxygen in space to achieve the reasonable coexistence and functional complementarity of three kinds of bacteria, which effectively improved the efficiency of the microbial consortia (Shahab et al., 2020).

During the degradation of complex compounds, the carbon source required for the growth of microbial consortia is generally a complex compound itself, but not all microorganisms in the microbial consortia can utilize complex compounds as carbon sources. A common solution is to construct sequential utilization patterns of substrates and intermediates. Applying this model can not only avoid substrate competition but also eliminate the negative feedback inhibition caused by some by-products (Park et al., 2020). However, this sequential utilization pattern does not have well-defined material and energy flow paths like most microbial consortia for synthesize compounds *de novo*. The material and energy flow pathways in microbial consortia for degrading complex compounds are more reticular in structure. Material and energy are transferred repeatedly between strains and may in any case be consumed rather than eventually pooling in a product. This complex interaction network of material and energy is beneficial for degrading complex compounds because it can make the structure of the microbial consortium more stable and more resistant to environmental fluctuations. In this microbial consortium, the relationship between strains becomes more complex as the number of strains increases. For microbial consortiums with many strains, it might be critical to consider higher-order interactions (HOIs) to ensure stable coexistence and function (Mayfield and Stouffer, 2017). For example, in a three-member consortium, a third population could attenuate the negative interaction between two antagonistic populations. The consortia can also be stable even if the third species is antagonistic to the two species so long as each population modulates the inhibitory interactions between the remaining two members (Kelsic et al., 2015). Thus, the presence of an additional population could synergize with an existing community, resulting in a more stable consortium. The HOIs can also extend to more population network topologies (Grilli et al., 2017). Furthermore, the importance of HOIs increases with the number of populations in the microbial consortium (Friedman et al., 2017).

Cross-feeding and quorum sensing (QS) are two commonly used artificial design approaches to maintain complex stability. Symbiotic relationships in microbial consortia with few strains are primarily based on single metabolite cross-feeding, such as an amino acid (Harcombe et al., 2018). For example, amino acid auxotrophies can create complex interdependencies between microorganisms. These relationships promote stability and robustness by allowing for metabolic redundancy among community members (Embree et al., 2015). However, the secretion of a single metabolite is often insufficient to support the normal growth of all strains in a big microbial consortium, limiting its robustness and stability. In a microbial consortium degrading complex compounds with many strains, developing a multiple-metabolite cross-feeding strategy is closer to the reality, which is used to strengthen the correlation between microbial entities. Central to this strategy is the selection of appropriate metabolic branches for cross-feeding, which involve multiple metabolites that are critical for cell growth and translocate across cell membranes. Amino acid anabolism and energy metabolism can often be selected to establish close cell–cell correlations resulting in a very stable co-culture system (Li et al., 2022). The social and gregarious behavior of single-celled organisms such as bacteria is usually accomplished through intercellular communication, which can occur through QS. QS primarily regulates collective features that involve energetically costly “public goods” and are most effective or even only functional if performed by a microbial consortium. Bacterial traits controlled by QS include genetic phenotypes, biofilm formation, promoting or inhibiting function, and virulence (Mashruwala et al., 2022; Pütz et al., 2022; Ramsay et al., 2022). QS even can be a driver and target of other functions (Striednig and Hilbi, 2022). However, one challenge in incorporating more members within microbial consortia that degrade complex compounds is that many QS systems are not completely orthogonal, and one solution is to design a new QS system. Recently, a sophisticated QS circuit with high dynamic ranges, low leakiness, and the ability to simultaneously regulate multiple sets of genes in 1 cell was designed and was used to autonomously and temporally regulate three metabolic fluxes involved in a pathway (Ge et al., 2022). This was a big step forward but not sufficient to deal with the more complex situation in microbial consortia. It was also discovered that QS systems can be used for cell–cell communication between distant populations (Luo X. et al., 2015). QS systems may play a key role in microbial consortia that degrade complex compounds, just as they now play an important role in synthetic microbial consortia with fewer strains, but they must be studied further.

## Current status of biodegradation of complex compounds by microbial consortia

Complex compounds are usually difficult to be efficiently degraded by natural microorganisms due to the complexity of their structures. Many researchers use microbial consortia to degrade complex compounds, especially common environmental pollutants. The current research progress on the degradation of complex compounds by microbial consortia is shown in Table 1. Common types of waste plastics such as polyethylene terephthalate (PET), polyethylene (PE), polystyrene (PS), and polyurethane (PU) have been degraded by microbial consortia. The study of strain interaction in natural microbial consortia is a necessary prerequisite for their construction. In fact, some isolated natural microbial consortia have the ability to degrade plastics. On this basis, researchers can add microorganisms to an isolated microbial consortium according to the relationships of the consortium to improve its efficiency, or build a simple microbial consortium to better understand degradability, gene regulation, or enzymatic activities. For example, about 90% of n-alkanes and aromatic hydrocarbons in petroleum hydrocarbons can be degraded by reconstructed microbial consortia (Bacosa and Inoue, 2015; Hu et al., 2020). Another obvious improvement is the treatment of sewage microbial consortia. Adding microalgae to activated sludge (a natural microbial consortium) can improve the adsorption and degradation efficiency of various compounds and even heavy metals in sewage (Sepehri et al., 2020). Alternatively, a microbial consortium can be constructed based on the interaction relationship between microorganisms, whose substrates are more targeted and generally can only degrade one or several specific types of complex compounds. At the same time, fewer strains are needed in the consortium, as each has its own clear mission. In artificial microbial consortia, the degradation process can be clearly represented and easily studied and even regulated. A representative example is the artificial two-strain consortium for CBP. One strain degrades complex cellulose into small molecular compounds, and another strain uses those small molecular compounds as substrates to synthesize the desired products (Wen et al., 2020). For complex compounds, especially pollutants in the environment (due to their refractory degradation and the complexity of the environment), the microbial consortium is one of the best choices for bioremediation.

**TABLE 1:** An overview of recent advances in the degradation of complex compounds by microbial consortia.

Substrate		Achievement	Co-culture strains	References
Plastic	PET	the weight loss of PET film reached 23.2% in 7 days	*Rhodococcus*, *Pseudomonas putida,* and two metabolically engineered *Bacillus subtilis* species	Qi et al. (2022)
	PE	demonstrated 81% ± 4% of weight reduction for LDPE strips over a period of 120 days	*Enterobacter* sp. bengaluru-btdsce01, *Enterobacter* sp. bengaluru-btdsce02, and *Pantoea* sp*.* bengaluru-btdsce03	Bardají et al. (2020)
	PS	demonstrated 12.4% of weight reduction PS, a weight loss of 23% of HIPS film in 30 days	*Bacillus spp.* and *Pseudomonas spp*.	Ho et al. (2018)
	PU	50.3% of proprietary aromatic PE-PU-A copolymer was consumed in 25 days	*Rhodobacterales, Rhizobiales, Burkholderiales, Actinomycetales, Sphingobacteriales*	Gaytán et al. (2020)
Petroleum hydrocarbons	n-alkane	synergistic rate of biodegradation of diesel oil was 85.54% ± 6.42%	*Pseudomonas stutzeri, Dietzia sp.*	Hu et al. (2020)
	Polycyclic aromatic hydrocarbons	nearly completely degraded fluorene and phenanthrene after 5 days	*Sphingomonas, Pseudomonas, Sphingobium, Dokdonella* and *Luteimonas*	Bacosa and Inoue, (2015)
Antibiotic		78.3% of Sulfonamide antibiotics had degraded after 4 weeks	*Firmicutes* and *Bacteroides*, represented by *Bacillus* and *Flavobacterium*	Liao et al. (2016b)
		All sulfamethoxazole (5 mg/L) had degraded in 3 h	nitrifying sludge (e.g., *Nitrosomonas, Dokdonella, Defluviicoccus, Pseudomonas, Zoogloea, Thauera,* and *Pseudomonas*)	Yan et al. (2022)
		After 28-day incubation at 25°C, the ciprofloxacin loss was nearly 100%	Classes *Gammaproteobacteria, Bacteroidia, Betaproteobacteria* and *Leucobacter*	Liao et al. (2016a)
Azo dyes		98.2% decolorization	A halophilic bacterial consortium from textile wastewater	Shi et al. (2021)
		All Orange II (250 mg/L) had been decolorization	*Vanrija humicola, Meyerozyma caribbica, Debaryomyces hansenii,* and *Meyerozyma guilliermondii*	Samir Ali et al. (2022)
Wastewater		NH_4_-N removal (100%) was observed within 7 days	*Chlorella vulgaris* and nitrifier-enriched-activated-sludge	Sepehri et al. (2020)
		the removal efficiency of acetoacetanilide (3200 mg/L) achieved 69.28 ± 0.42% within 14 days	*Paenarthrobacter, Rhizobium, Rhodococcus, Delftia* and *Nitratireductor*	Zhang et al. (2023)
Lignocellulose		produced 3.94 g/L butanol, which was five times higher than the control	C. *cellulovorans* and C. *beijerinckii*	Wen et al. (2020)
		7.61 g/L of butanol was generated from untreated corncob	*Thermoanaerobacterium thermosaccharolyticum* and *Clostridium acetobutylicum*	Jiang et al. (2020)
		0.44 g/g bioethanol production in biological pretreatment of the lignocellulosic cotton stalk	*Saccharomyces cerevisiae* YPH499 and *Pachysolen tannophilus* 32691	Malik et al. (2021)

### Degradation of petroleum hydrocarbons by microbial consortia

Petroleum hydrocarbons are important energy resources and raw materials for all walks of life. Petroleum hydrocarbon pollutants, such as normal paraffin, cycloalkanes, and aromatics, are recalcitrant compounds and are listed as priority pollutants (Holliger et al., 1997; Costa et al., 2012; Sajna et al., 2015; Li et al., 2020; Qian et al., 2021). They can usually be degraded in the presence of several natural microorganisms, each of which can decompose a specific set of molecules. Thus, microbial consortia have advantages in crude oil bioremediation (Zanaroli et al., 2010). Microbes in oil-contaminated areas adapt to the environment, resulting in genetic mutations in offspring that enable them to degrade petroleum hydrocarbon compounds (McDonald et al., 2006; Varjani and Upasani, 2016). Many novel species of microorganisms such as *Anaerobaculum*, *Desulfacinum infernum*, *Methanococcus thermolithotrophicus*, *Thauera phenylacetica*, and *Geobacillus subterraneus* have been isolated from sites of contamination (Garcia and Oliveira, 2013).

The top-down approach is often used to construct microbial consortia for the degradation of petroleum hydrocarbons. The degradation efficiency can be improved or the substrate range can be broadened by adding new strains to natural microbial consortia. The bioremediation capacity of microbial consortia in oil-contaminated areas is often limited due to the poor biodiversity of native microbial consortia, where the presence of microorganisms with complementary substrate specificities to degrade different hydrocarbons is lacking (Ron and Rosenberg, 2014). Microbial consortia with the potential to degrade petroleum hydrocarbon compounds can be screened out from oil-contaminated areas, before improving the degradation ability or substrate extensiveness of the microbial consortia system by artificial compounding or adding artificially engineered bacteria. There are reports showing that microbial consortia are superior to single bacteria in utilizing hydrocarbon contaminants in petroleum crude oil as the sole carbon source (Varjani et al., 2013). Such consortia show an increased degradation rate of diesel and polycyclic aromatic hydrocarbons (PAHs) when cultured under laboratory conditions (Varjani and Upasani, 2013). Ibrar and Zhang (2020) constructed a microbial consortium containing *Lysinibacillus, Paenibacillus, Gordonia,* and *Cupriavidus spp*. that could produce biosurfactants to enhance the ability of other bacteria to degrade petroleum hydrocarbons. Their results showed that the microbial consortia could use common polycyclic aromatic hydrocarbon pollutants (naphthalene and anthracene) as the sole carbon source. Therefore, artificial microbial consortia systems are a potential research direction to improve bioremediation efficiency in oil-contaminated areas (Varjani et al., 2015).

**FIGURE 2:**
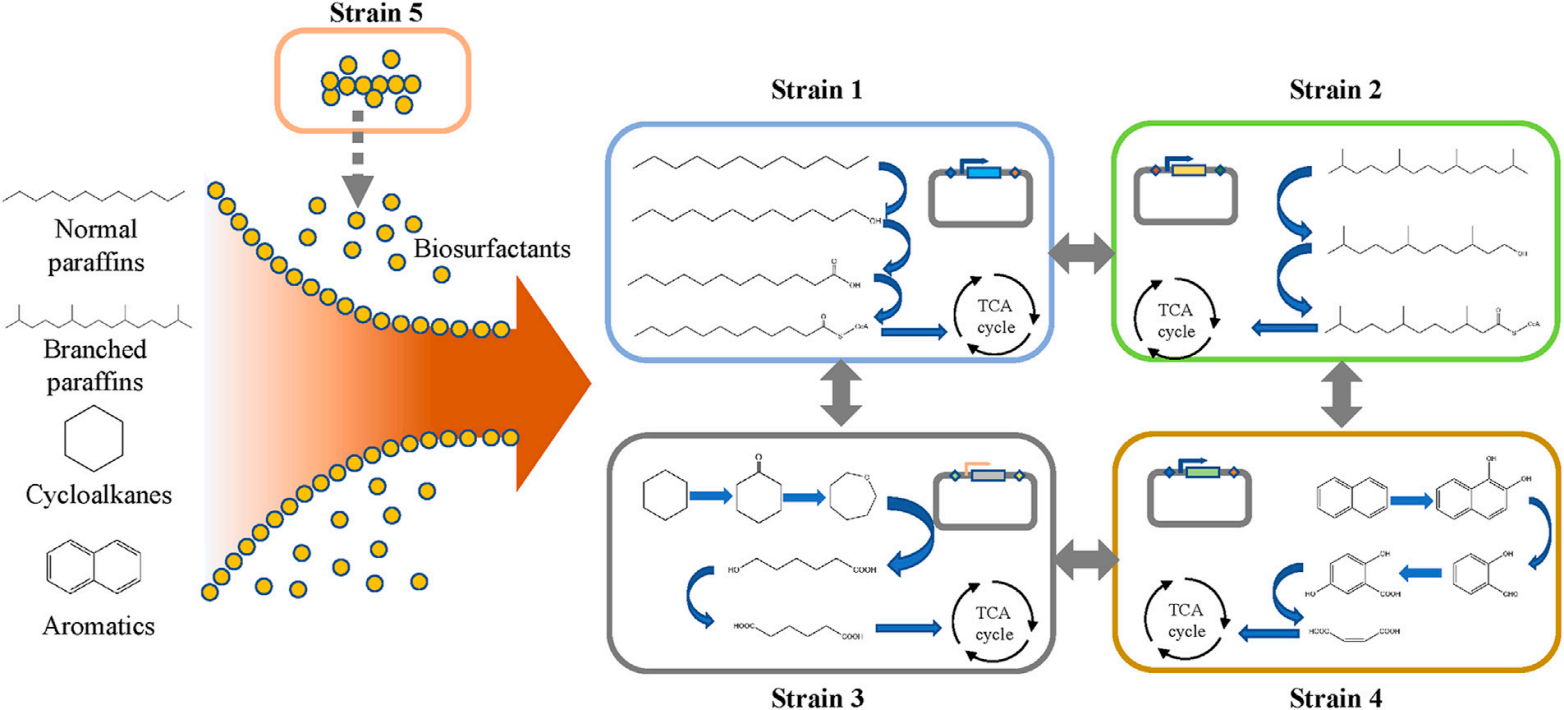
Biodegradation of petroleum hydrocarbons.

The bottom-up approach can also be used to construct microbial consortia based on the degradation pathway of petroleum hydrocarbons, which has been elucidated in the literature. As shown in Figure 2, the biodegradation of petroleum hydrocarbon can be divided into several processes (Li and Ding, 2021). In the first step, microorganisms enhance the bioavailability of petroleum hydrocarbon pollutants by chemotactic movements and secreting surfactants (Ahmad et al., 2020). These surface-active materials increase the surface area and bioavailability of hydrophobic and water-insoluble substrates, thereby increasing the speed at which petroleum hydrocarbons can approach microorganisms. Then, the petroleum hydrocarbons enter the cell through the transport process, mainly by free diffusion, passive transport, active transport, and endocytosis (Gu et al., 2016). Finally, the petroleum hydrocarbon is degraded in the cell. The degradation pathways of petroleum hydrocarbon compounds mainly include aerobic degradation and anaerobic degradation. Common pathways for the degradation of linear alkanes include the initial degradation of alkanes and the oxidation of methyl groups, leading to the formation of alcohols, followed by the dehydrogenation of aldehydes to form their corresponding carboxylic acids. Then, the fatty acids are metabolized by the β-oxidation pathway (Abbasian et al., 2015). The degradation of cycloalkanes and aromatic hydrocarbons is more difficult than that of linear alkanes, as the former needs to be sequentially opened by hydrolase or isomerase and then degraded through degradation pathways that are different from those of linear alkanes (Gupta et al., 2015; Ghosal et al., 2016; Dhar et al., 2020; Li and Ding, 2021). Furthermore, each degradation pathway of petroleum hydrocarbons is relatively long, which will bring greater growth pressure to cells. Under the condition of ensuring degradation efficiency, a single strain cannot undertake all the functions of petroleum hydrocarbon degradation and biosurfactant production at the same time. Therefore, many studies are using microbial consortia to degrade petroleum hydrocarbons, where different bacterial species undertake different functions in the degradation process. This can not only reduce the growth pressure of individual cells but also improve the tolerance of bacterial groups to harsh environments through cooperation between different bacterial species, thereby making the entire degradation system stable and robust.

The degradation efficiency of petroleum hydrocarbons can be improved by modifying engineered bacteria according to the degradation pathway. Such genetically modified engineered microorganisms can degrade or assist in the degradation of complex compounds. Luo Q. et al. (2015) constructed oil biodegradation bacteria to promote the biodegradation of diesel. The alkane hydroxylase (alkB) gene was introduced into *Escherichia coli*, giving it the ability to degrade diesel fuel. The diesel-induced expression of the AlkB protein increased the diesel degradation rate from 31% to 50% after 24 h. Enhancing surfactant production is conducive to improving the accessibility of petroleum hydrocarbons to the strains, which is beneficial for improving their degradation efficiency. Wu et al. (2018) engineered *B. subtilis 168* by integrating surfactant synthesis activators, knocking out competing pathways, and enhancing the supply of fatty acid precursors, resulting in a significant increase in surfactant yield. Furthermore, different bacteria with auxiliary functions and petroleum hydrocarbon-degrading bacteria can be combined to form a consortium.

There is a way to improve the ability of the microbial consortia which is by adjusting the interspecific relationship of microbial consortia. Shuang et al. (2019) constructed a three-bacteria system with, a significantly improved degradation efficiency of phenanthrene obtained through the synergistic effect between the bacterial species. Ghorbannezhad et al. (2018) created a microbial consortium using eight fungi, three yeasts, and four bacteria, and an oil degradation assay for various combinations, including a bacterial mixed culture, a fungal mixed culture, a fungal-bacterial mixed culture, and a sequential fungal-bacterial mixed culture. The experimental results showed that the repair effect of the synergistic microbial consortia was generally significantly higher than that of a single strain. The results demonstrate that communication between different microorganisms in the microbial consortia may improve the degradation ability of petroleum hydrocarbons.

As one of the advantages of a microbial consortium, strains with additional functions can be added without affecting the degradation pathways of complex compounds. The most economically valuable strategy is to add a microbial that can use degradation products as carbon sources or substrates for the biosynthesis of high-value products. In addition, the depletion of degradation products favors the forward progression of the degradation pathway. Thus, the synthesis of high-value products increases the economic benefits of microbial consortia for degrading complex compounds (Wang et al., 2022).

### Degradation of plastics by microbial consortia

A large amount of plastic is produced globally every day, but only 21% of plastics are recycled or incinerated, and most of the remainder is discarded or buried, greatly polluting the environment (Law, 2017). Under natural conditions, the whole process of plastic degradation requires a timeframe of more than 50 years (Webb et al., 2013). Plastic waste can be degraded through physical processes, chemical processes, or biodegradation (Andrady, 2011). Microbial degradation has been increasingly studied due to its safety, rapidity, and low cost. Many plastics are biodegraded by microbial consortia rather than individual strains, possibly because of the limited metabolic capacity of individual microorganisms (Qi X. et al., 2021). Yu et al. (2019) found that microorganisms in a consortium had higher biodegradation efficiency than individual strains because the potentially toxic intermediates can be removed by other microorganisms present.

Many have investigated the degradation mechanism of microbial consortia constructed by top-down approaches. Vargas-Suárez et al. (2019) selected microbial consortia from degraded foam blocks collected in landfills. They found that in the presence of microbial consortia, the carbon utilization efficiency of their strain was more efficient in degrading multiple types of complex plastics than when it was independent because of the interspecific interaction. To elucidate the mechanism by which landfill microbial consortia attack PU plastics, Gaytán et al. (2020) investigated the degradation of a microbial consortium selected from a municipal landfill, which was able to disperse PU in water as the sole carbon source of growth. The study showed that the degradable enzyme gene of selected microbial consortia has great potential in the direction of bioremediation. After understanding these cooperative relationships, microbial consortia can be constructed to achieve plastic degradation.

**FIGURE 3:**
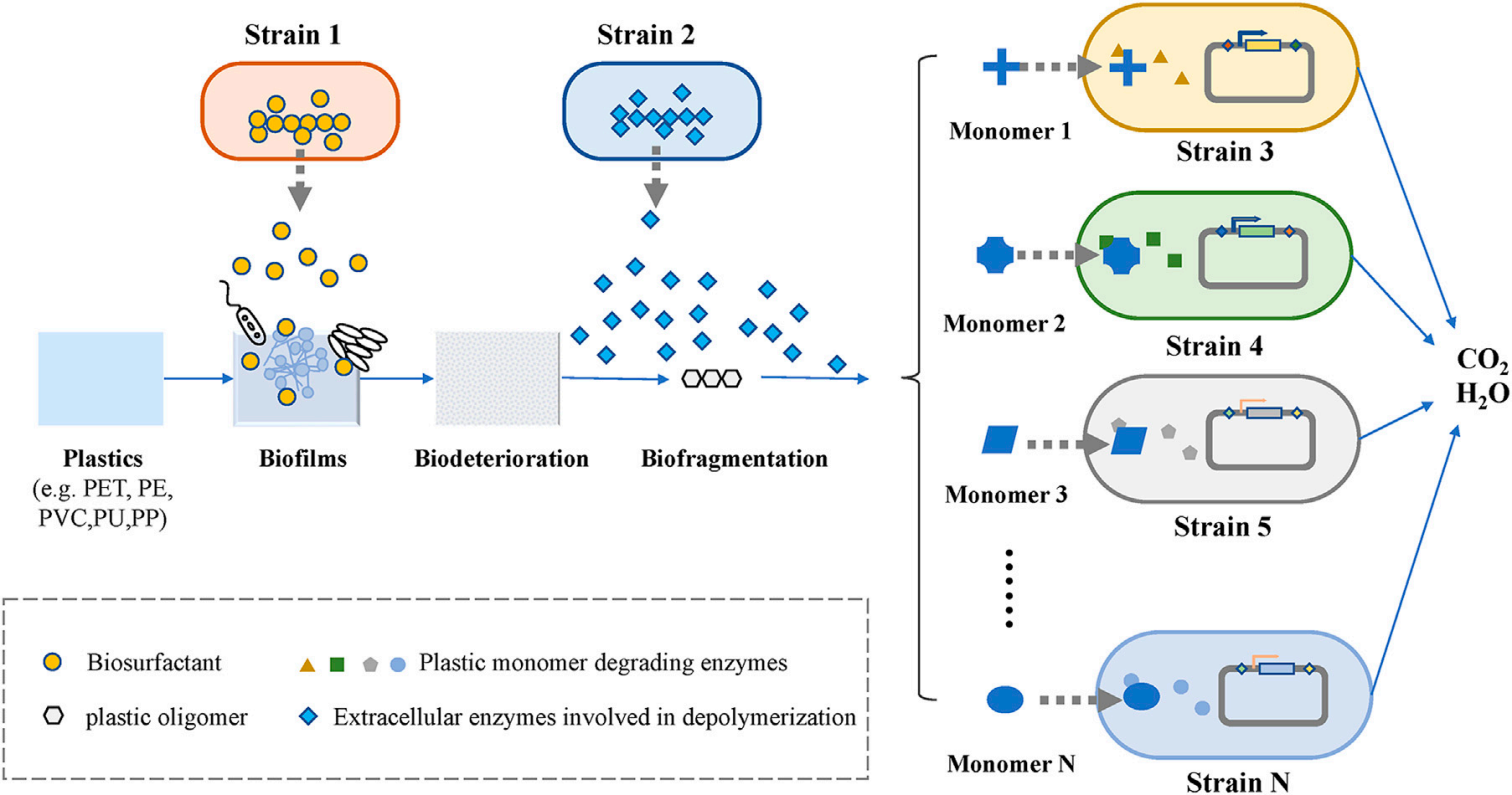
Biodegradation of plastics.

The bottom-up approach can be used to construct microbial consortia based on the different degradation pathways of plastics, whose steps are relatively clear. As shown in Figure 3, the whole process of microbial degradation can be summarized into three stages: biodeterioration, biofragmentation, and biodegradation (Zhang et al., 2022). The biodeterioration stage refers to the degradation of plastic polymer surfaces by biofilms that are formed (Ru et al., 2020). In general, biofilms of microbial consortia exhibit a better ability to degrade plastics than those of single bacteria at this stage. Due to the natural hydrophobicity of plastics, it is necessary to introduce hydrophilic functional groups on the surface of plastics to facilitate the attachment of microorganisms (Nauendorf et al., 2016). For example, the biosurfactant-producing module in the microbial consortium system for petroleum hydrocarbon degradation described above can also be used in microbial consortia for plastic degradation. Tribedi et al. (2015) demonstrated that biofilm-promoting compounds, such as mineral oil and surfactants for biofilm attachment, enhanced the biodegradation of plastics. Fungi can also play an important role in the degradation of plastics, as they can attach to plastic surfaces *via* their hyphae and provides an attachable platform for other microorganisms (Sánchez, 2020). Biofragmentation is a depolymerization step that convert plastic polymers into smaller units by the action of extracellular enzymes and free radicals (Jenkins et al., 2019). Plastic-degrading enzymes are divided into two broad categories: extracellular enzymes and intracellular enzymes. These different groups of enzymes have been found to act similarly to microbial laccases, peroxidases, lipases, esterases, and cutinases (Gan and Zhang, 2019), and they are mainly involved in depolymerizing the long carbon chains of plastic polymers to form mixtures of oligomers, dimers, and monomers. Subsequently, these monomers are then processed by different strains. Once these plastic monomers are successfully transported into cells, they undergo a series of enzymatic reactions that lead to their complete degradation into oxidative metabolites (Ho et al., 2017). The complete degradation of plastic requires the participation of a variety of enzymes, and the enzymes required for the degradation of different plastics are different. Therefore, microbial consortia are a suitable choice to increase the rate of plastics degradation, especially for a mixture of various plastics.

For artificial microbial consortia constructed by bottom-up approaches, the consortium can indirectly improve biodegradation through metabolic cross-feeding or the production of metabolites that induce co-metabolic degradation (Hu et al., 2020). The degradation pathways of plastics can be divided into different modules according to key rate-limiting enzymes and intermediates, and each module can be assigned to different strains. These strains constitute the initial microbial consortium. This approach has the advantage of reducing the metabolic burden of each strain and increasing the tolerance of the microbial community to harsh environments through strain-to-strain interactions. Qi X. H. et al. (2021) constructed a microbial consortium consisting of *Rhodococcus*, *Pseudomonas putida,* and two engineered *B. subtilis* species for the degradation of PET. The two engineered *B. subtilis* secrete PET hydrolase and monohydroxyethyl terephthalate hydrolase to achieve the initial degradation of plastics. Then, *R. jostii* and *Pseudomonas putida* were added to degrade terephthalic acid and ethylene glycol. The final microbial consortia could completely degrade 23.2% of a PET film at room temperature. Furthermore, as a result of in-depth research on the degradation pathways of synthetic plastics, it has also become possible to build a microbial community degradation platform to degrade and convert synthetic plastics into high-value products (Qi et al., 2022; Sullivan et al., 2022).

### Degradation of antibiotics and azo dyes by microbial consortia

Antibiotics have been widely used as an effective class of effective drugs, and their presence has been reported in sewage treatment plant effluent, sewage treatment plant biosolids, surface water, groundwater, and drinking water (Barancheshme and Munir, 2018; Zhang et al., 2018). Such antibiotic contamination has posed a major global threat. Some scholars claim that future bioremediation work will focus on enzymatic remediation, and biotechnology should be prioritized over chemical treatment to minimize contamination after treatment (Kumar et al., 2019). Microbial consortia showed excellent degradability in studies on the biodegradation of antibiotics. There are many types of antibiotics, and each antibiotic biodegrades in different ways. Some antibiotics are so complex that they require the cooperation of several strains to be completely degraded. Thus, microbial consortia also have advantages in degrading antibiotics.

Using the top-down approach to construct microbial consortia for the degradation of antibiotics is common at present. *Firmicutes* and *Bacteroides*, represented by *Bacillus* and *Flavobacterium*, are the main bacteria in sulfa-degrading consortia. These microbial consortia obviously can degrade sulfonamides, and almost half of the antibiotics can be degraded after 1 week, with an average degradation rate of 78.3% after 4 weeks (Liao et al., 2016b). Activated sludge is a common research object in natural microbial consortia for the degradation of antibiotics at present. Many researchers have used bacterial liquids in the activated sludge of sewage treatment plants to conduct experiments aiming to study the degradation characteristics and influencing factors of antibiotics degradation. For example, two microbial consortia isolated from activated sludge were constructed to degrade sulfamethoxazole (Larcher and Yargeau, 2011). And their degradation rates increased after sulfamethoxazole was pretreated with ozone (Larcher and Yargeau, 2013). Dominant bacteria for antibiotic degradation were screened out from the activated sludge, and a dominant microbial consortium was constructed to degrade the drugs, which included *Microbacterium sp. BR1, Rhodococcus sp. BR2, Achromobacter sp. BR3, Ralstonia sp. HR1, Ralstonia sp. HR2* and *Tsukumurella sp. HR3*. The microbial consortia degraded sulfamethoxazole with a mineralization rate of 58.0% ± 1.3% (Bouju et al., 2012).

Based on the above research results, a bottom-up approach can be used to construct artificial microbial consortia. Su et al. (2018) built a microbial consortium containing *Streptomyces sp.* and *Bacillus licheniformis* with a high degradation capacity toward β-cypermethrin, where 88.3% of β-cypermethrin could be removed within 72 h. Further, the results of the artificial microbial consortia have excellent stability and can be used in environmental restoration. Wu et al. (2020) constructed a co-culture system that could be applied to actual sewage for bioremediation, which degraded more than 80% of the tetracycline after 10 days.

Azo dyes are the most widely used synthetic dyes in textile and garment printing and dyeing. In the production and use processes of the dyes, about 10%–15% is discharged into the environment without treatment, which seriously affects the health of the contacts. These dyes have a strong solubilizing ability in water and are difficult to be removed by traditional approaches (Lellis et al., 2019). There are many approaches to treating azo dye sewage, among which microbial decolorization is considered to be the most effective and environmentally friendly. The first step in the bacterial degradation of azo dyes is to destroy the azo bonds in the dye molecules. The decolorization of azo dyes by fungi begins with hyphal adsorption, followed by the secretion of extracellular enzymes to break chemical bonds. Azo bond cleavage reactions can occur both extracellularly and intracellularly, which is favorable for the cooperation of microorganisms to degrade azo dyes. Most importantly, a single microorganism will produce toxic aromatic amines during the degradation of azo dyes, whereas microbial consortia will not (Joshi et al., 2008). This is why such a decolorization approach with microbial consortia is promoted.

However, the underlying molecular mechanism of synergistic metabolism in the microbial consortia system has not been revealed. Therefore, the current microbial consortia are mainly constructed by the top-down approach. Shanmugam et al. (2017) explored this mechanism through molecular biotechnology, finding that their microbial consortia system could biodegrade and mineralize azo dyes to a higher degree due to the synergistic relation and division of labor in the consortia. Microbial consortia have also shown excellent performance in practical applications of azo dye degradation. For example, Selim et al. (2021) isolated 21 fungi that could degrade azo dyes from contaminated soil. Textile sewage treated with microbial consortia systems can be used to irrigate non-edible plants and alleviate the global water shortage. Although the use of azo dyes has been limited, their environmental impact is still serious. Therefore, the ability of microbial consortia to degrade azo dyes deserves further study and contributes to environmental protection stategy.

### Treatment of sewage by microbial consortia

With the rapid development of modern industrialization and economic globalization, the large amount of sewage discharged by various industries is becoming a serious global environmental problem (Kong et al., 2018; Sierra et al., 2018). Traditional treatment systems are usually expensive, demand massive amounts of energy, and are often still incapable of solving all challenges associated with sewage. Using microbial consortia to treat sewage is a relatively clean and efficient approach, especially in the treatment of sewage eutrophication (Plöhn et al., 2021). The microbial consortia often studied for wastewater treatment are bacto-algae consortia developed from activated sludge. Microalgae can switch autotrophic and heterotrophic metabolism depending on the availability of carbon sources and nutrients in the surrounding environment. Therefore, microalgae are a popular candidate for building microbial consortia in water (Raja et al., 2008; Kumar et al., 2010; Subashchandrabose et al., 2013; Wijffels et al., 2013; Li et al., 2016). Microbial consortia for wastewater treatment constructed by the top-down approach have showed good stability, but with less screening process than the bottom-up approach. Therefore, they will not be discussed much here.

For microbial consortia constructed by the bottom-up approach, the combination of microalgae and bacteria showed a beneficial promoting effect. The oxygen and carbon dioxide in algae and bacteria is beneficial for growth, where algae secretions are the main carbon sources (carbohydrates, proteins, and fats) for bacteria. The metabolites of bacteria can be used as promoters for algae growth. In addition, the cell surface of microalgae can provide a stable habitat for bacteria (Ramanan et al., 2016). Bacteria break down organic matter into mineral forms and secrete extracellular metabolites such as auxin and vitamin B12, which are necessary for the growth of microalgae (Salim et al., 2014). Thus, compared with individual microorganisms, those combined with microalgae are more efficient in detoxifying organic and inorganic pollutants and removing nutrients from sewage (Subashchandrabose et al., 2013; Wijffels et al., 2013; Xiong et al., 2017). One study experimentally compared the arsenic accumulation and transformation of *Chlorella vulgaris, Aspergillus oryzae*, and bacto-algae pellets under different concentrations of arsenic and phosphorus. Among all the treatments, the removal efficiency of the bacto-algae ball was the highest and its ability to accumulate arsenic was the strongest (Li B. et al., 2019). Similarly, constructed microbial consortia have shown advantages in treating wastewater eutrophication. Mujtaba et al. (2017) studied the simultaneous removal of nutrients (ammonium and phosphate) and COD in a co-culture system of *Chlorella vulgaris* and *P. putida*. They found that the removal of nutrients and COD by the co-culture system was higher than that of each individual culture system, indicating that the nutrient absorption capacity of *Pseudomonas* putida was improved in the consortia. Thus, the combined use of microalgae and microbial consortia has broad prospects in sewage treatment.

### Consolidated bioprocessing by microbial consortia

Consolidated bioprocessing (CBP) is considered one of the most potent and cost-effective ways to produce biofuels and other high-value products. It can complete the production of lignocellulose-degrading enzymes, the hydrolysis of lignocellulose, and microbial fermentation in one step. However, it is difficult to find a suitable microorganism to produce all the enzymes required for the degradation of lignocellulose and the production of high-value-added products. A promising alternative is bioprocessing based on microbial consortia. Most of the current approaches for constructing CBP microbial consortia are bottom-up approaches due to their remarkable controllability.

For the microbial consortia constructed by the bottom-up approach, some experiments verified that microbial consortia can indeed improve the efficiency of CBP. Zuroff et al. (2013) found that consortia of *C. phytofermentans* and *S. cerevisiae* produced ethanol from α-cellulose more efficiently than monocultures. If an artificial microbial consortium is constructed according to the division of labor among strains, the efficiency of CBP could be greatly improved. For example, the microbial consortia of *Zymomonas mobilis* and *Candida tropicalis* can convert enzymatically hydrolyzed lignocellulosic to ethanol with a yield reaching 97.7% (Patle and Lal, 2007). In addition to the production of ethanol, CBP can be used to produce other compounds such as halomethanes and lactic acid. Bayer et al. (2009) used a microbial consortium of engineered yeast and the cellulolytic bacterium *Actinotalea fermentans* to produce halomethanes from raw switchgrass, corn stover, bagasse, and poplar. Shahab et al. (2018) assembled an artificial microbial consortium of the cellulolytic enzyme-secreting aerobic fungus *Trichoderma reesei* with facultative anaerobic lactic acid bacteria. The results showed that the theoretically maximal lactic acid yield was obtained in the experiment.

Many experimental results show that substrate degradation efficiency is the rate-limiting step in CBP. Therefore, it is necessary to increase the substrate degradation rate to further provide higher monosaccharide concentrations. The activity of enzymes in microbial consortia may be additionally activated and improved, thereby improving the efficiency of the CBP system (Kuhar et al., 2015). To further investigate the factors affecting the secretion of degradative enzymes in microbial consortia, Puentes-Tellez and Salles, 2018 applied a reductive screening approach based on molecular phenotype, identification, and metabolic characterization to select the desired microbial consortia. They found a minimally active microbial consortium with efficient lignocellulose-degrading ability. The degradation potential of the least active microbial consortia reached 96.5%. The enhanced degradation efficiency of lignocellulose by the mixed bacteria was more obvious in another experiment, which established a microbial consortium of *Serratia sp.* and *Arthrobacter sp.* to improve cellulose degradation. The enzymatic activity was increased by 30%–70% after co-cultivation. In addition, the degradation rate of the microbial consortia was increased by more than 30%. In another application direction, the use of microbial consortia with ligninolytic degradation ability can significantly increase the lignocellulose degradation rate in a fixed fluidized bed reactor (An et al., 2022). When the problem of the degradation efficiency of lignocellulose is solved, the construction of microbial consortia becomes much clearer. When isolated cellulose-degrading microbial consortia were co-cultured with *Clostridium acetobutyricum*, the utilization rate of cellulose was greatly improved, and a relatively high butanol product concentration was obtained (Wang et al., 2015).

## Conclusion

Most current research on complex-compound-degrading microbial consortia has focused on native microbial consortia isolated from the environment. However, we believe that artificial microbial consortia are the direction of future research. With the help of metabolic engineering and synthetic biology, the construction of microbial consortia systems shows a strong degradation potential, which serves a new approach for the efficient utilization of complex substrates and the remediation of the environment. Although the mechanism of intercellular communication in large microbial consortia is still unclear, and the regulatory means are imperfect, it is predicted that with the deepening of the relevant research, the strong metabolic capacity and robustness of artificial microbial consortia will promote their use in the field of degrading complex compounds.
